# Buffalo Academy of Medicine

**Published:** 1908-08

**Authors:** Irving M. Snow, Dewitt H. Sherman, J. H. Potter, N. G. Russell, L. Schroeter


### Buffalo Academy of Medicine

**Report of Milk Committee.**

IN 1907, Dr. Allen A. Jones, President of the Buffalo Academy of Medicine, appointed a milk committee consisting of Drs. Snow, Sherman, Russell, Julius H. Potter and Schroeder.

This committee begs to report that there has recently arisen in American communities a keen and intelligent interest in the cleanliness and nutrient qualities of cow's milk. It is appreciated that the health of adults and especially of young children is much influenced by the purity of the milk supply and that serious illness and often death may be the result of taking infected milk. The principal dangers of bad or dirty milk are:

1. The spread of typhoid fever, scarlet fever, gastrointestinal disease, and the like.

2. The numerous cases of bovine tuberculosis among the cattle of Western New York.

3. The sale of milk deficient in solids and the use of preservatives and thickeners in milk and cream.

The supervision of the milk supply of New York is under the control of two official boards, namely, (1) the State Agricultural Board, which deals directly with the sanitary condition of the cattle, dairies and farms; (2) the Health Officers of the various cities and villages who watch the distribution of milk to the consumer.

The functions of the State Agricultural Board are as yet imperfectly developed. There is practically no inspection for bovine tuberculosis. The tuberculous cattle from the west whose sale is prohibited in New England or Europe are dumped into Western New York.

There should certainly be state legislation forbidding the importation into New York State of cattle untested for tuberculosis. Again, the dairy farms of the state should be frequently patrolled, the farmers should be instructed in cleanliness, in aerating and cooling the milk. The milk producer should be forbidden to sell milk when their families and employees are suffering from any communicable disease. No water from a polluted stream or well should be used about a dairy. In some of the cities of the state most scientific and progressive work is accomplished chiefly through frequent chemical and bacteriologic examination, by inspection of the dairy farms, and by admonishing and penalising a careless or dishonest farmer or milkman.

The Milk Committee of the Buffalo Academy of Medicine has conferred together and with the Health Commissioner, who have mutually agreed that the need of the community is a clean raw milk.

Immediate measures for the improvement of the milk supply of Buffalo as agreed by the health commissioner, Dr. Ernest Wende, and the committee are, (1) to keep the milk cool in summer by the use of refrigerator cars as is done in New York City. Dr. Wende is planning to confer with the railroad officials on this subject. If the corporations refuse to furnish refrigerator cars, to transport milk in hot weather, a statement of existing conditions and an appeal for relief should be made to the Public Service Commission; (2) to make very frequent examinations, say three hundred per month, of the milk, for bacteria, total solids and preservatives. Bacteriological examinations are already made. The health office has been granted three additional inspectors of food and drugs; one of these, a trained chemist, is to work exclusively with milk.

The frequent examinations of milk will give a true index to its quality and cleanliness and there will be no reason why the citizens cannot be served with a clean raw milk. It would be desirable to give the utmost publicity to the condition of the city milk. A dirty or dishonest farmer or dairyman should be punished by either forbidding him to sell his milk in the city for a limited time, or by revoking his license.

(Signed) Irving M. Snow,
Dewitt H. Sherman,
J. H. Potter,
N. G. Russell,
L. Schroeter.

